# Spectral detector computed tomography imaging of histologically confirmed splenic pathologies in 30 canine patients: a comparison of virtual non-contrast images and true unenhanced images

**DOI:** 10.3389/fvets.2025.1645439

**Published:** 2025-09-25

**Authors:** Alkje M. van Gemmeren, Lydia K. Claußen, Philipp Lietz, Sebastian Meller, Adriano Wang-Leandro, Andreas Beineke, Verena Nerschbach, Holger A. Volk, Kristina Merhof

**Affiliations:** ^1^Department of Small Animal Medicine, University of Veterinary Medicine Hannover, Foundation, Hannover, Germany; ^2^Institute of Pathology, University of Veterinary Medicine Hannover, Foundation, Hannover, Germany

**Keywords:** spectral detector computed tomography, dual-energy, virtual-non-contrast technique, true unenhanced images, splenopathy

## Abstract

**Introduction:**

Spectral detector computed tomography (SDCT), a cutting-edge technique in veterinary medicine, offers various options for characterizing soft tissues. One such option is the virtual non-contrast (VNC) algorithm, which reconstructs pre-contrast images from post-contrast spectral data by identifying and subtracting iodine pixels. This method has demonstrated accuracy in assessing abdominal organs in healthy canine patients. To determine whether this algorithm can be applied in a clinical setting to reduce radiation dose, scan time, and, consequently, the duration of general anesthesia, its accuracy in patients with pathology must be evaluated.

**Methods:**

Our study compared the Hounsfield units (HUs) measured in VNC and true unenhanced (TUE) images for splenic pathologies in general, as well as for specific types of pathologies based on their imaging characteristics. All differences were determined through subtraction and evaluated by using cut-off values of ≤ 5, ≤ 10, and ≤ 15 HUs. Differences in HUs of ≤ 10 were deemed negligible, while differences ≤ 15 HUs were considered acceptable. Two one-sided t-tests were performed. Additionally, image quality and iodine subtraction were evaluated using a five-point Likert scale.

**Results:**

For splenic pathologies in general, lesions classified by imaging characteristics and various pathologies, the VNC technique demonstrated high efficiency in contrast medium subtraction and exhibited a strong agreement with TUE images regarding their HUs. In 305 drawn regions of interest (ROIs), the differences in HUs between TUE and VNC were ≤ 15 HUs in 98.0%, ≤ 10 HUs in 93.1%, and at least 66.2% were below the limit of 5 HUs. SDCT images provided better image quality than conventional computed tomography images.

**Discussion:**

Our study suggests that VNC images reconstructed from post-contrast SDCT data may serve as a promising alternative to the standard use of a pre- and post-contrast series. The VNC images provide high quality and reliability in imaging structurally altered splenic tissue, but show a potential weakness in the calculation of some mineralized lesions.

## 1 Introduction

Conventional computed tomography (CT) scans are commonly used as a diagnostic tool for canine patients with splenic pathologies. These examinations assist clinicians and owners in deciding on further diagnostics and treatment options. Previous veterinary studies utilizing conventional CT have detailed the imaging characteristics of splenic pathologies (1–9). Spectral detector computed tomography (SDCT) offers new opportunities for characterizing soft tissue pathologies, as demonstrated in numerous studies within human medicine (10–18). SDCT is based on the simultaneous measurement of low and high-energy-spectrum photons at the detector level, utilizing a standard polyenergetic X-ray beam. This additional information provides insights into tissue composition while still permitting the calculation of conventional CT images from the same data. Recently, SDCT has become increasingly accessible in veterinary medicine, although research is still in its early stages (18–21). Certain tissues absorb high-energy photons similarly, but show different absorption values for low-energy photons due to their specific binding energy of the k-shell electron. This is, for example, true for iodine and bone, which display overlapping Hounsfield units (HU) in conventional CT. The virtual non-contrast (VNC) imaging technique, derived from spectral CT data, uses this information to identify and subtract iodine pixels from post-contrast scans to generate virtual pre-contrast series. The VNC technique aims to provide similar information to the true native scan and does not intend to enhance the diagnostic capabilities of CT examinations regarding pathologies.

As noted in earlier human studies, the HUs from the VNC series corresponded well with those from the true unenhanced (TUE) images in normal organs (13–15). The VNC technique has also been used to evaluate diseased organs, like the liver, urogenital tract, adrenal gland, gastrointestinal tract, and peripheral arteries (10, 12, 17, 22–28), and some authors already see the possibility that in certain diseases, such as kidney masses, the TUE scan can be omitted (29, 30).

In progenitor studies at our institution, we examined the equivalence of VNC and TUE series in the abdominal organs of healthy dogs (19) and rabbits (21). The results supported previous findings from human medicine, showing negligible differences between VNC and TUE images. Since there are no existing studies in human or veterinary medicine regarding splenic pathologies, we aimed to determine whether the VNC algorithm would be equally effective in dogs with structurally altered spleens. If the VNC technique proves reliable across various organs and pathologies, the pre-contrast scan could potentially be omitted in future procedures. This would reduce radiation exposure by half, shorten scan times, and decrease the time under general anesthesia. Such benefits are particularly important for clinically critical patients or those with higher anesthetic risks. Additionally, shorter scan times would help minimize stress in patients who need to be scanned awake, like polytrauma patients or rabbits (21).

Our aim was to determine the equivalence of the VNC technique to TUE images by comparing the HUs calculated by the VNC algorithm with those in the TUE images of splenic pathologies in dogs. We utilised detailed imaging characteristics of splenic pathologies to deliberately place regions of interest (ROIs). We aimed to ascertain whether certain types of lesions, based on their imaging characteristics, as well as specific pathologies, influence the precision of VNC values to evaluate if this technique could be applicable in a clinical setting. Our first hypothesis was that the reliability of the VNC technique compared to TUE images is generally high in splenic pathologies, particularly for certain types of lesions and across various pathologies. Our second hypothesis was that the subjective image quality of SDCT is superior to that of conventional CT images.

## 2 Materials and methods

### 2.1 Selection and description of study population

For this retrospective study, the medical records of all canine patients who underwent a diagnostic workup for various symptoms at the Clinic for Small Animals of the University of Veterinary Medicine Hannover Foundation between November 2021 and March 2023 were reviewed. This included a SDCT examination of the abdomen (Philips IQon Spectral CT, Philips Health Care Germany). The inclusion criteria were splenic lesions visible in SDCT and a histopathological examination of these lesions. Samples were collected through splenectomy or ultrasound-guided Tru-Cut biopsies.

From all dogs that met the inclusion criteria, data on breed, sex, age at the time of imaging, weight, and reason for initial presentation were collected using simple calculation software (Microsoft® Excel, Version 16.0). Since the data used was part of routine clinical work, no approval from an ethics committee was required.

### 2.2 Data recording and analysis

#### 2.2.1 CT-imaging

All SDCT examinations were conducted under general inhalation anesthesia with Isoflurane CP® (CP-Pharma Handelsgesellschaft mbH, Burgdorf, Germany) and performed in sternal recumbency with the head-first position. The CT protocol included one native scan followed by a venous phase series after the patient received 2 mL/kg of the iodine-based contrast medium (ICM) Xenetix® 350 (Guerbet GmbH, Sulzbach, Germany) intravenously, which corresponds to 70 mg iobitridol/kg through a Power-Dual Injector System (MEDRAD Stellant/Bayer HealthCare, Leverkusen, Germany) without an additional saline flush. Image acquisition for the subsequently analyzed contrast-enhanced CT (CECT) venous phase began after a 60-s delay following positive feedback from the bolus tracking software (trigger at 150 HUs), with the ROI located in the thoracic aorta on the level of the heart base immediately caudal to the arcus aortae. If bolus tracking failed, the scan was manually initiated 60 to 70 s after the start of contrast medium administration. All SDCT scans were performed with the following parameters: maximum tube potential 120 kV, automatic mAs depending on the standard protocol of different patient weight categories (range 174–320 mAs), and a pitch of 0.6, with a gantry rotation time of 0.5 s, a slice thickness of 2 mm, and a 512 images matrix. A standardized CT-protocol for the abdomen was applied, using an appropriate soft tissue window (window level: 60; window width: 350) and bone window (window level: 800; window width: 2,000).

#### 2.2.2 SDCT-analysis

##### 2.2.2.1 Characterization of imaging features

A board-certified radiologist (KM), unaware of the histopathology results, evaluated all available CT studies by considering several imaging characteristics related to structurally altered splenic tissue. She documented the presence and degree of peritoneal effusion, as well as the size and structure of regional lymph nodes. Splenic alterations were categorized as diffuse, focal, or multifocal.

The following characteristics were documented for focal and multifocal lesions: the number of lesions; their localization; shape; borders; margination; extent; size of the largest lesion in all three planes; capsule formation; cavitation; attenuation compared to the surrounding splenic parenchyma before and after the application of contrast medium; enhancement pattern and degree of contrast enhancement; and presence of mineralization (Table 1).

**Table 1:** The imaging features evaluated to characterize the splenic lesions.

Parameter	Possible rating
Peritoneal fluid	None, mild, moderate, severe
Surface of the spleen	Smooth, irregular
Contours of the spleen (apart from the lesion)	Sharp angulation, rounded borders
Lesion type	Diffuse, focal, multifocal
Number of lesions	1, 2–5, 6–10, > 10
Localization of lesions	Head, body, tail, head and body, body and tail, head and tail, all three areas
Shape of lesions	Round, oval, amorphous, different types of shapes if multifocal lesions
Border of lesions	Irregular, regular
Margination of lesions	Well-defined, ill-defined, different for different lesions
Extent of lesions	Intraparenchymal, extending over splenic border, both types of lesions present
Size of lesions	Maximum extension in cm (applies to the largest lesion, measured in all three planes)
Capsule formation	Absent, present
Cavitation	Absent, one lesion, several lesions
Attenuation pre/ post contrast compared to surrounding parenchyma	Hypoattenuating, isoattenuating, hyperattenuating, different types of attenuation within the same lesion or in several lesions
Enhancement pattern	Homogeneous, heterogeneous, mainly peripheral, circular peripheral (target lesions), different types of enhancement in different lesions
Degree of enhancement	Mild, moderate, severe, different within the same lesion or in different lesions
Mineralization	Absent, mild, moderate, severe
Size of splenic lymph nodes	Normal, mild/moderate/severe enlargement
Structure of splenic lymph nodes	Homogeneous, heterogeneous
Enhancement pattern of splenic lymph nodes	Homogeneous, heterogeneous

##### 2.2.2.2 Objective assessment

Images were retrospectively evaluated by a board-certified radiologist (KM), a radiologist in training (PL), and a clinical veterinarian (AVG) using system-associated software certified for medical imaging analysis (IntelliSpace Portal Version 11.x / Philips Healthcare) on a monitor approved for imaging review. The examiners were permitted to adjust window width and level to enhance lesion visibility. Initially, the TUE images were paired with auto-registration post-processing software to align them with the three reconstructions derived from post-contrast data: Conventional, VNC, and monoenergetic (MonoE) 70 keV. In cases where the auto-registration process failed, manual adjustments along the x-axis were performed. Blinding for the different series was unnecessary as the VNC and TUE series are easily distinguishable.

**Figure 1:**
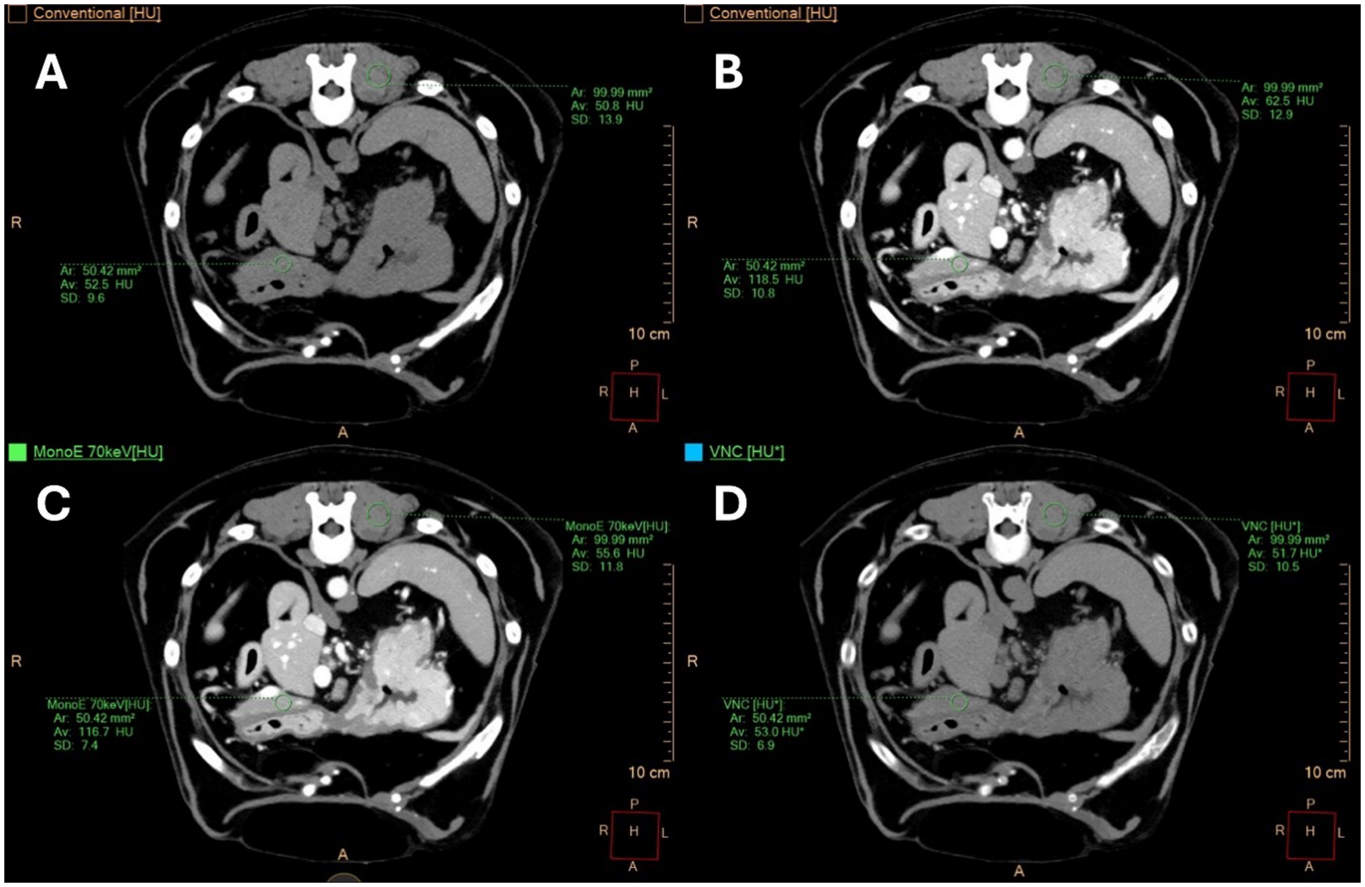
Transverse computed tomography (CT) images of the abdomen of a twelve-year-old spayed male Labrador Retriever at the level of the right pancreatic limb. **(A)** Shows conventional images, **(B)** shows post-contrast conventional images, **(C)** presents monoenergetic (MonoE) images at 70 keV post-contrast, and **(D)** displays virtual non-contrast (VNC) images. A 1 cm^2^ region of interest (ROI) is placed in the paraspinal musculature, while another ROI with the largest possible diameter is positioned in the pancreas.

For all patients, the paraspinal muscles, pancreas (Figure 1), and gallbladder were used as reference organs, with one ROI defined for each. The paraspinal muscle and pancreas showed the best results concerning the accuracy of HUs in VNC images compared to TUE images in our progenitor study (19). The gallbladder was additionally chosen because its content should have no contrast uptake, and there should be no significant difference in HUs between VNC and TUE images. Larger differences in HUs between VNC and TUE images for muscle and pancreas compared to the previous study, and larger differences regarding the gall bladder content, could indicate a technical error.

**Figure 2:**
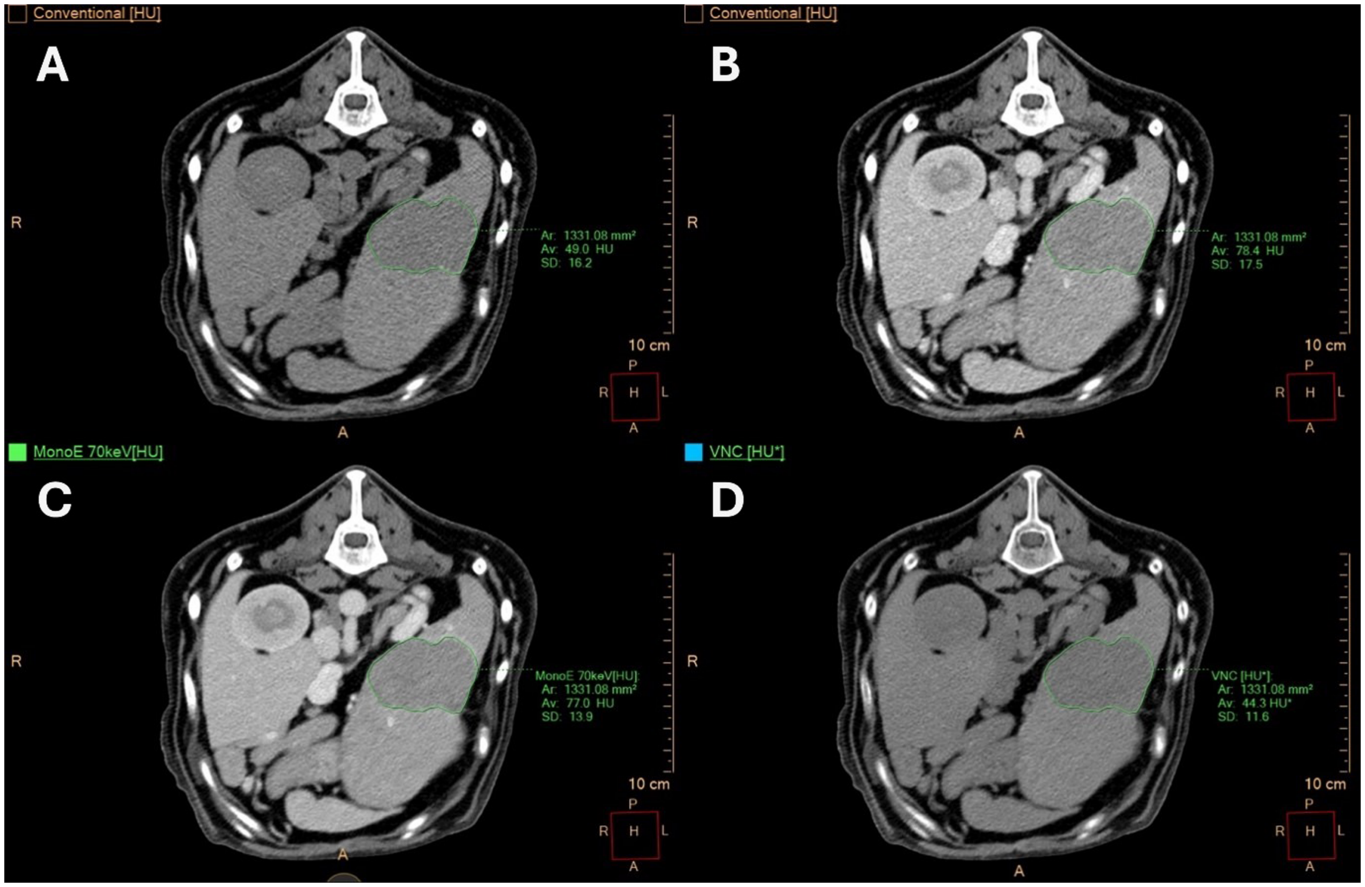
Transverse computed tomography (CT) images of a twelve-year-old male Standard Poodle with a focal splenic lesion. **(A)** Shows true unenhanced (TUE) images, **(B)** displays conventional images post-contrast, **(C)** presents monoenergetic (MonoE) images at 70 keV post-contrast, and **(D)** exhibits virtual non-contrast (VNC) images. A polygonal region of interest (ROI) was positioned at the largest transverse cross-section of the lesion. Histopathology revealed a hematoma in the patient.

Depending on the type of lesions, ROIs in the spleen were distributed as follows:

1. Diffusely altered spleens: two ROIs each in the splenic tail, head and body.
2. Spleens with a single focal lesion: one polygonal ROI encompassing the entire lesion in the transverse plane (Figure 2), two ROIs each in the periphery and center of the lesion, and two ROIs situated in normal-appearing splenic tissue outside the lesion.
3. Spleens with multifocal lesions: one polygonal ROI in the largest lesion in the transverse plane, two ROIs each in the periphery and center of the lesion, and two in normal-appearing splenic tissue outside the lesions. In cases of multiple small lesions of equal size, one ROI has been drawn for each.

In cases of mineralized lesions, a polygonal ROI and two additional ones each in the periphery and center were established. For all ROIs, the corresponding HU was measured in TUE, VNC, post-contrast, and monoenergetic sequences. A size of 100 mm^2^ was selected for each ROI, except for the polygonal ROI, which encompassed the entire lesion. When lesions were smaller than 100 mm^2^, the largest possible ROI was used.

##### 2.2.2.3 Subjective assessment

A radiologist in training (PL) and a board-certified radiologist (KM), both blinded to the histopathology results, performed the subjective analysis in consensus. The VNC and TUE series were automatically paired and positioned side by side. The examiners were permitted to adjust the window width and level to optimize lesion visibility. The patients were randomly ordered for review by the clinical veterinarian (AVG). The parenchymal iodine subtraction (Table 2) and the image noise and quality (Table 3) were evaluated using a previously established five-point Likert scale (19). The iodine subtraction was examined at six locations: the pancreas, gallbladder, spinal muscle, liver, spleen, and the primary lesion.

**Table 2:** A five-point Likert scale for assessing parenchymal iodine subtraction in virtual non-contrast (VNC) images compared to true unenhanced (TUE) images by evaluating the removal of contrast medium (CM).

Parenchymal iodine subtraction in VNC compared to TUE	
1	Insufficient subtraction of CM
2	Partly sufficient removal of CM with larger, incomplete areas
3	Moderate removal of CM with incomplete areas in parts of parenchyma
4	Almost complete removal of CM
5	Complete removal of CM

**Table 3:** A five-point Likert scale to assess image noise and quality in spectral-based images compared to conventional images.

Image noise and quality of spectral based images compared to conventional images	
1	Spectral based images markedly worse than conventional images
2	Spectral based images mildly worse than conventional images
3	Spectral based images equivalent to conventional images
4	Spectral based images mildly better than conventional images
5	Spectral based images markedly better than conventional images

### 2.3 Histopathological samples

All histopathological samples were acquired through ultrasound-guided Tru-Cut biopsy or total splenectomy during laparotomy at the Clinic for Small Animals of the University of Veterinary Medicine Hannover Foundation and were subsequently fixed in 10% buffered formalin. Soft tissue semi-automatic biopsy needles with adjustable penetration depth (BIO CORE 2, 16 G (1.67 mm x 10 cm), HVM Medical Products GmbH, Fulda, Germany) were used for the biopsies. The location for the Tru-Cut biopsies was selected based on ultrasound imaging and depended on the decision of the ultrasonographer. The number of biopsies taken depended on the appearance of the lesions and the quality of the samples obtained (2–3 per patient). Due to the retrospective nature of the study, the location and number of Tru-Cut biopsies varied and were not standardized.

All tissue samples underwent examination at the Institute for Pathology of the University of Veterinary Medicine Hannover Foundation by Diplomates of the European College of Veterinary Pathologists (ECVP). The samples were dehydrated in a graded series of ethanol and then embedded in paraffin wax. From these samples, 2–3 μm thick slices were prepared and stained with hematoxylin–eosin. Subsequently, these histopathological preparations were examined under a light microscope at up to 100x magnification according to ECVP standards and were classified into the following groups based on their predominant findings: nodular hyperplasia, extramedullary hematopoiesis, hemorrhage, thrombosis, hemosiderosis, neoplasia, and inflammation. If more than one pathological finding was present, for instance, hemangiosarcoma and hemosiderosis, the patients were classified according to the predominant and clinically more relevant diagnosis, which in this case was categorized as hemangiosarcoma. All recorded findings were entered into the patient’s records.

### 2.4 Statistical method

Appropriate software (Microsoft Excel 2019; GraphPad Prism 10.2.2) was utilized for the statistical evaluation of the collected data. Initially, the differences in HU-values of corresponding VNC and TUE images of each ROI for reference organs, normal spleen, and splenic lesions were determined through subtraction (19) and verified by establishing cut-off values of ≤ 5, ≤ 10, and ≤ 15 HUs based on veterinary and human medical studies (13–15, 17, 19). Values with a difference in HUs of ≤ 10 were deemed negligible, while differences between 10 and 15 HUs were considered acceptable (13, 14). The cut-off value of ≤ 5 HUs was also included to assess and illustrate the potential accuracy of VNC images. A two one-sided *t*-test (TOST) was performed to test the equivalence of TUE and VNC series, utilizing an opposing null hypothesis that mean differences between TUE and VNC were greater than 5, 10, or 15 HUs, respectively. To emphasize the analysis of various lesions and pathologies, all measured ROIs for each respective category were grouped and analyzed independently of the number of dogs.

Subsequently, multiple comparisons of the mean differences were conducted on various imaging characteristics to assess their influence on the accuracy and comparability of VNC with TUE images, using a one-way analysis of variance (ANOVA) test, including Tukey’s multiple comparisons test for *p*-value correction. An unpaired t-test was performed to evaluate the influence of the location of regions of interest situated at the center or the periphery of focal lesions. For testing normal distribution, the D’Agostino and Pearson test was performed. Simple column bar graphs and box-and-whisker plots were created. Summarised data were reported as mean ± standard deviation (SD). A *p*-value of less than 0.05 was deemed significant. All tests were two-sided.

## 3 Results

### 3.1 Study population

In total, 30 dogs met the inclusion criteria. The affected breeds included the following: Rhodesian Ridgeback (4), Golden Retriever (3), Labrador Retriever (3), American Staffordshire Terrier (1), Appenzeller Mountain Dog (1), Beagle (1), Fox Terrier (1), French Bulldog (1), Galgo Español (1), Standard Poodle (1), Magyar Vizsla (1), Nova Scotia Duck Tolling Retriever (1), Whippet (1), and 10 crossbreeds. Fourteen dogs were female (of which eleven were spayed), while sixteen were male (also eleven were spayed). The median age of the dogs at presentation was 11 years, ranging from 4.1 years to 13.2 years. The median weight was 26.3 kg, ranging from 4.8 kg to 50.4 kg.

Eighteen of the 30 patients were initially presented for general staging due to other findings, such as prostatopathy or neoplasia, in which a structurally altered spleen was a secondary finding. Five patients were referred to the clinic with acute abdomen, and seven patients exhibited lethargy, where a splenic lesion was the primary finding.

### 3.2 Histopathological findings

Twenty-eight histopathological samples were obtained following a splenectomy, and two samples were collected via a tru-cut biopsy under ultrasonographic guidance. The histopathological examination revealed ten malignant pathologies: five hemangiosarcomas, four lymphomas, and one metastatic anal sac carcinoma, along with 18 benign lesions, which included nine nodular hyperplasias, four cases of extramedullary hematopoiesis, three hematoma, one thrombus in the splenic body, and one instance of hemosiderosis. There were no cases of splenitis or splenic abscessation. Two splenic samples were unremarkable, one being a Tru-Cut biopsy and the other a splenectomy.

Hemosiderosis was a common finding in 14 cases of both benign and malignant splenic pathologies, while extramedullary hematopoiesis was noted as an additional finding in four benign cases and one malignant case.

### 3.3 Characterization of splenic lesions

Among the 30 spleens examined, 21 displayed multifocal changes, seven had focal changes, and two exhibited diffuse changes. Twelve patients had free peritoneal fluid (mild: 3/30, moderate: 6/30, severe: 3/30). Of the 21 cases of multifocal splenopathies, 13 patients had more than 10 lesions, four had between 6 and 10 lesions, and four had between 2 and 5 lesions. The lesions were categorized based on their location: splenic head: 2/30, splenic body: 2/30, splenic tail: 4/30, head and body: 1/30, body and tail: 1/30, all three regions: 20/30.

Four patients exhibited partial mineralization in at least one of their lesions (mild: 2/30, moderate: 1/30, severe: 1/30). Capsule formation was only observed in one lesion. Eleven patients presented lesions with cavitation (8/30 in one lesion, 3/30 in multiple lesions). The attenuation of lesions pre-contrast compared to the surrounding tissue was hypoattenuating in 15, isoattenuating in six, and hyperattenuating in two patients. Five patients displayed different types of attenuation within the same lesion or across several lesions. In the post-contrast series, eight of the 28 patients with focal or multifocal lesions showed hypoattenuation of lesions compared to surrounding tissue, eleven patients had hyperattenuating lesions, and nine patients exhibited different types of attenuation within the same lesion or in multiple lesions. The enhancement pattern of splenic lesions, or the entire spleen in diffuse cases, was homogeneous in nine patients and heterogeneous in 13 patients. Seven patients demonstrated various enhancement patterns across different lesions, with three of these patients having one target lesion each. One spleen showed no enhancement at all. The maximum extent of the splenic lesions ranged from 0.7 cm to 18.2 cm, measured across all three planes.

### 3.4 Objective assessment

A total of 305 ROIs were drawn, of which 215 were located in the splenic parenchyma. This resulted in 610 Hounsfield unit values (in VNC and TUE series) that were statistically evaluated.

The differences in HUs between TUE and VNC for all measured ROIs were ≤ 15 HUs in 98.0%, ≤ 10 HUs in 93.1%, and at least 66.2% were below the limit of 5 HUs.

**Figure 3:**
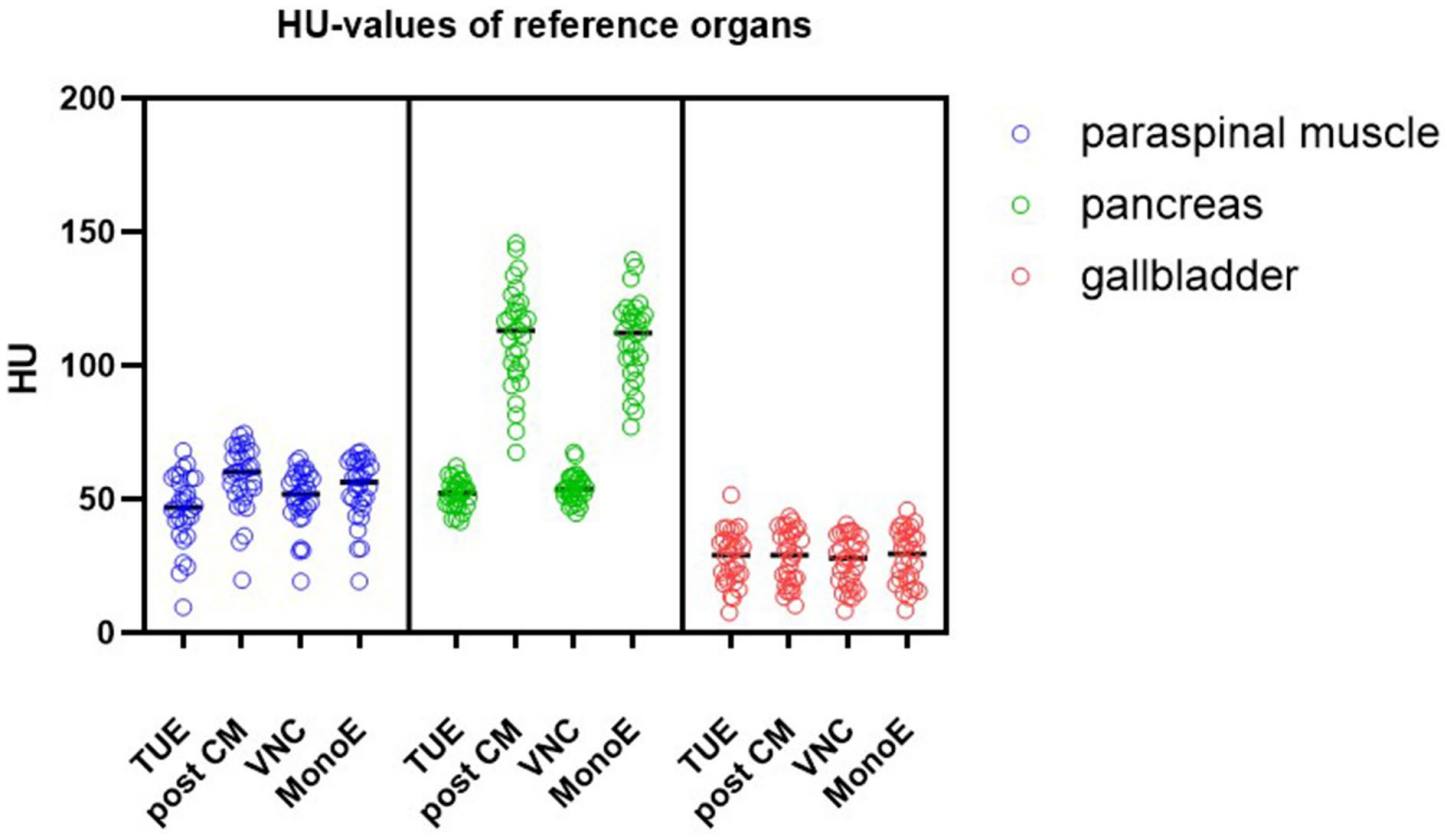
The distribution of Hounsfield units (HUs) in conventional true-unenhanced (TUE) images, conventional post-contrastmedium (post CM) as well as in virtual non-contrast (VNC) images and post-contrast monoenergetic (MonoE) images at an energy level of 70 keV in reference organs.

The values for the reference organs exhibited a strong correlation between VNC and TUE images, as well as conventional post-contrast images with MonoE images at an energy level of 70 keV (Figure 3).

**Figure 4:**
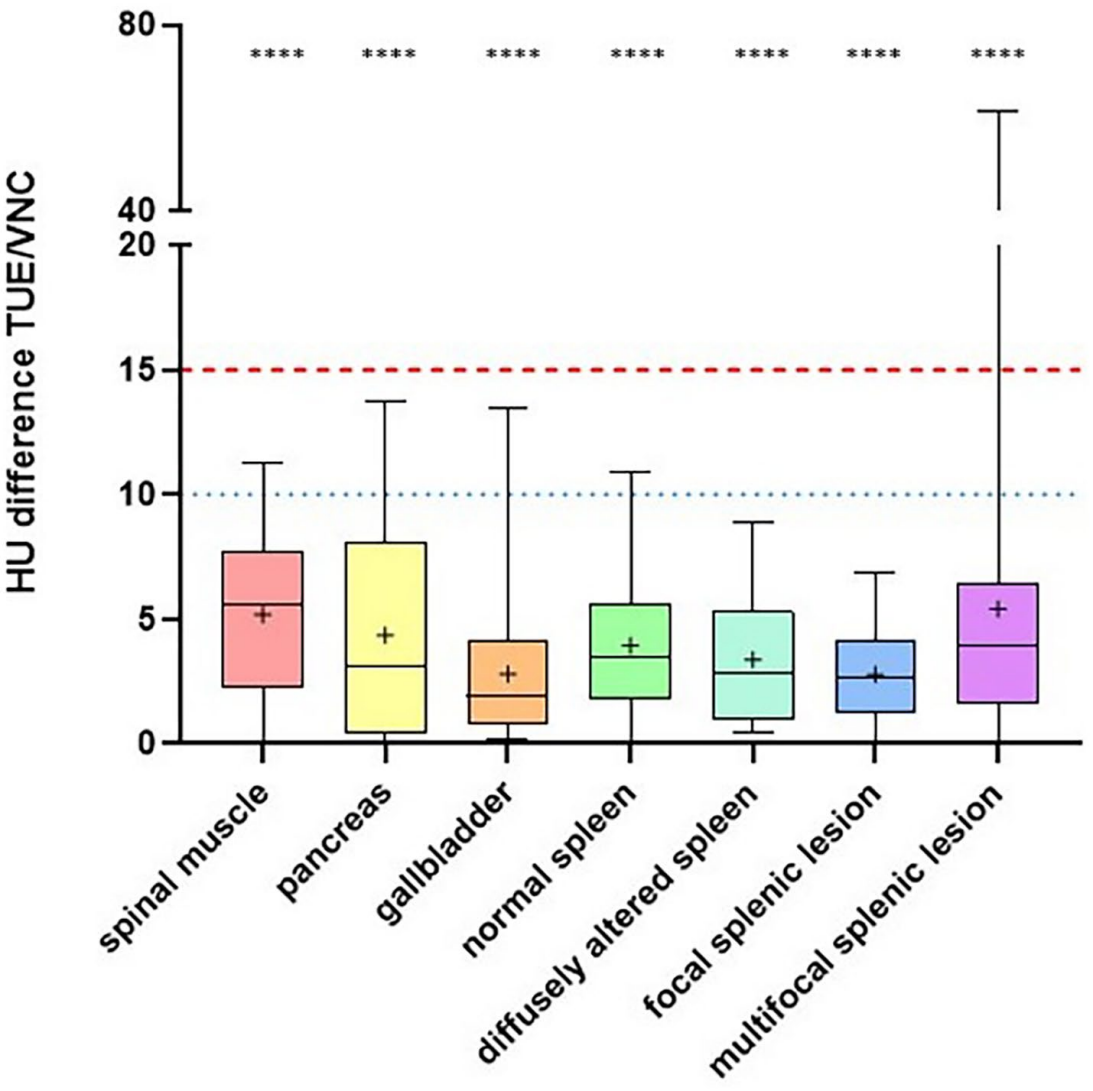
The differences in Hounsfield units (HUs) between true unenhanced (TUE) and virtual non-contrast (VNC) images for all regions of interest (ROIs). 98.0% of the differences in HUs between VNC and TUE were within the acceptable limit of ≤ 15 HUs (red dashed line), while 93.1% remained under the limit of ≤ 10 HUs (blue dotted line), which was considered negligible. The median of each box is represented as a horizontal line. The lower and upper margins of each box indicate the 25th and 75th percentiles. The cross indicates the mean. The results of the TOST (Table 5) within each group of organs, was extremely significant (****p < 0.0001) for the threshold of < 10 HU difference, which was considered a negligible difference.

For the reference organs and normal splenic tissue, 100% of the calculated differences in HUs between TUE and VNC were below the threshold of 15 HUs. In diffusely altered spleens and those with focal lesions, the differences between TUE and VNC were below the threshold of 10 HUs in 100% of cases. In spleens with multifocal lesions, the differences were ≤ 15 HUs in 94.7% and ≤ 10 HUs in 91.2% of 114 drawn ROIs (Figure 4). Regarding their histopathological diagnoses, patients with hemangiosarcoma (5), lymphoma (4), metastatic carcinoma (1), nodular hyperplasia (9), hematoma (3), and thrombus (1) showed that 100% of the evaluated differences between TUE and VNC were ≤ 10 HUs. Among patients with extramedullary hematopoiesis (4), 81.5% had differences ≤ 15 HUs, and 66.7% had differences ≤ 10 HUs. In one patient with hemosiderosis as the primary histopathological finding, 75% of measured differences between TUE and VNC were ≤ 10 HUs. Categorized into the cut-off values of ≤ 5, ≤ 10, and ≤ 15 HUs, the differences in HUs between TUE and VNC images for the reference organs, various groups regarding their imaging characteristics, and the splenic pathologies are listed in Table 4. The collected data showed predominantly a Gaussian distribution.

**Table 4:** Displays the results for the predefined cut-off values of ≤ 15, ≤ 10, and ≤ 5 Hounsfield units (HUs) in both percentage and total counts of regions of interest (ROIs).

Region of interest	Cut-off values for the difference between TUE and VNC					
	≤ 15 HUs		≤ 10 HUs		≤ 5 HUs	
All organs (30)	98.0%	299/305	93.1%	284/305	66.2%	202/305
Reference organs (30)						
Spinal muscle	100%	30/30	90%	27/30	43.3%	13/30
Pancreas	100%	30/30	83.3%	25/30	66.7%	20/30
Gallbladder	100%	30/30	96.7%	29/30	90.0%	27/30
Normal splenic tissue	100%	56/56	96.4%	54/56	64.3%	36/56
Imaging characteristics						
Diffusely altered spleens (2)	100%	12/12	100%	12/12	75%	9/12
Splenic head	100%	4/4	100%	4/4	50%	2/4
Splenic body	100%	4/4	100%	4/4	75%	3/4
Splenic tail	100%	4/4	100%	4/4	100%	4/4
Focal splenic lesions (7)	100%	33/33	100%	33/33	97%	32/33
Polygonal ROI	100%	7/7	100%	7/7	100%	7/7
Periphery of lesion	100%	14/14	100%	14/14	92.9%	13/14
Center of lesion	100%	12/12	100%	12/12	100%	12/12
Multifocal splenic lesions (21)	94.7%	108/114	91.2%	104/114	57%	65/114
Polygonal ROI	95.2%	20/21	95.2%	20/21	61.9%	13/21
Periphery of lesions	95.2%	40/42	92.9%	39/42	61.9%	26/42
Center of lesions	94.4%	34/36	86.1%	31/36	47.2%	17/36
Multiple small lesions	93.3%	14/15	93.3%	14/15	60%	9/15
Histopathological diagnosis (30)						
Hemangiosarcoma (5)	100%	25/25	100%	25/25	72%	18/25
Lymphoma (4)	100%	24/24	100%	24/24	66.67%	16/24
Metastatic carcinoma (1)	100%	5/5	100%	5/5	100%	5/5
Nodular hyperplasia (9)	100%	50/50	100%	50/50	78%	39/50
Hematoma (3)	100%	13/13	100%	13/13	61.54%	8/13
Extramedullary hematopoiesis (4)	81.48%	22/27	66.67%	18/27	29.63%	8/27
Thrombus (1)	100%	4/4	100%	4/4	100%	4/4
Hemosiderosis (1)	75%	3/4	75%	3/4	50%	2/4
Considered normal on histopathology (2)	100%	7/7	100%	7/7	85.71%	6/7

Differences in HUs between true unenhanced (TUE) and virtual non-contrast (VNC) images of ≤ 10 were considered negligible, while differences of ≤ 15 were still deemed acceptable. The total number of patients is shown in brackets.

The two one-sided t-tests conducted to evaluate the equivalence of TUE and VNC regarding the limits of ≤ 15, ≤ 10, and ≤ 5 HU differences, aimed at supporting the previously mentioned results, indicated a strong agreement between TUE and VNC images across all ROIs (*p* < 0.0001 for differences ≤ 10 HUs). However, the group with splenic lesions due to extramedullary hematopoiesis showed no significant equivalence of HUs in the TUE and VNC series, with a p-value of 0.0693 for differences ≤ 15 HUs. All results of the TOST are illustrated in Table 5.

**Table 5:** Presents the results of the two one-sided *t*-tests (TOST) conducted to examine the equivalence of virtual non-contrast (VNC) and true unenhanced (TUE) images.

Region of interest	*p*-values for differences ≤ 15 HUs	*p*-values for differences ≤ 10 HUs	*p*-values for differences ≤ 5 HUs
All ROIs (*n* = 305)	< 0.0001	< 0.0001	0.0994
Spinal muscle (*n* = 30)	< 0.0001	< 0.0001	0.6239
Pancreas (*n* = 30)	< 0.0001	< 0.0001	0.2081
Gallbladder (*n* = 30)	< 0.0001	< 0.0001	0.0001
Spleen in total (*n* = 215)	< 0.0001	< 0.0001	0.2798
Normal spleen (*n* = 56)	< 0.0001	< 0.0001	0.0024
Type of lesion			
Diffusely (*n* = 12)	< 0.0001	< 0.0001	0.0264
Focal (*n* = 47)	< 0.0001	< 0.0001	< 0.0001
Multifocal (*n* = 156)	< 0.0001	< 0.0001	0.7349
Histopathology			
Hemangiosarcoma (*n* = 35)	< 0.0001	< 0.0001	< 0.0001
Lymphoma (*n* = 32)	< 0.0001	< 0.0001	0.0003
Metastatic carcinoma (*n* = 7)	< 0.0001	< 0.0001	0.0075
Nodular hyperplasia (*n* = 64)	< 0.0001	< 0.0001	< 0.0001
Hematoma (*n* = 19)	< 0.0001	< 0.0001	0.6138
Extramedullary hematopoiesis (*n* = 35)	0.0693	0.6798	0.9904
Thrombus (*n* = 6)	< 0.0001	< 0.0001	0.0823
Hemosiderosis (*n* = 6)	0.0032	0.0566	0.7314
without findings (*n* = 11)	< 0.0001	< 0.0001	0.0132

A *p*-value of less than 0.05 indicates that the Hounsfield unit (HU) values for VNC and TUE are equivalent. The total number of regions of interest (ROIs) is provided in brackets.

Only two patients had differences between the TUE and VNC values of more than 15 HUs. One patient, who had four small soft tissue attenuating lesions, exhibited a difference of 15.2 HUs in one of these lesions. The remaining three lesions in this spleen showed differences below the threshold of 10 HUs. This patient had hemosiderosis on histopathology.

**Figure 5:**
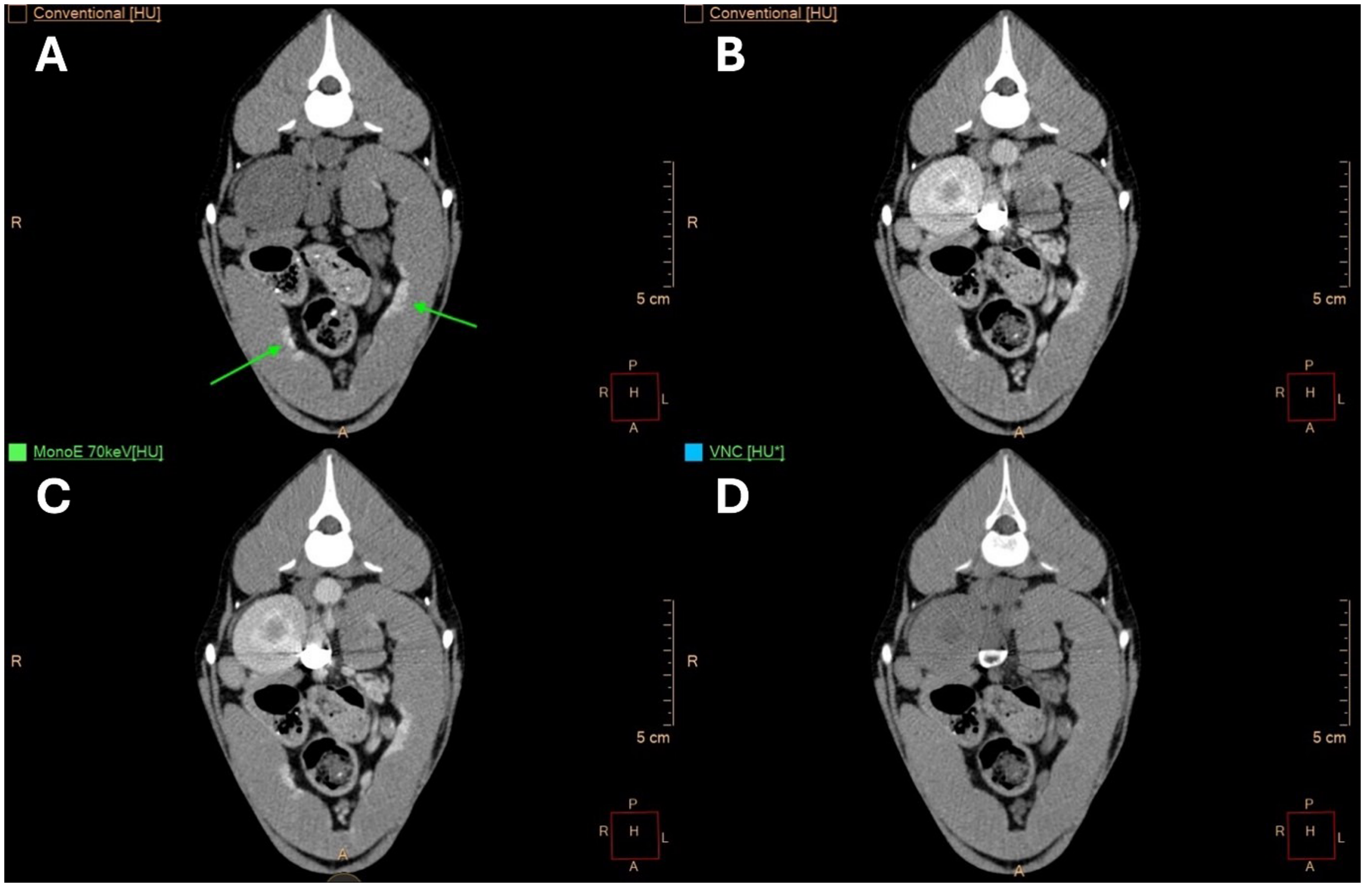
Transverse computed tomography (CT) images of an eleven-year-old, male, spayed Whippet at the level of the right kidney show multiple moderately mineralized lesions at the splenic hilus (green arrows) and additional mineralized lesions in a more central location (not included in this image plane). **(A)** Displays true unenhanced (TUE) images, **(B)** shows conventional images post-contrast, **(C)** presents monoenergetic (MonoE) images at 70 keV post-contrast, and **(D)** displays virtual non-contrast (VNC) images. The algorithm has mainly removed mineralization in the VNC images **(D)**.

**Figure 6:**
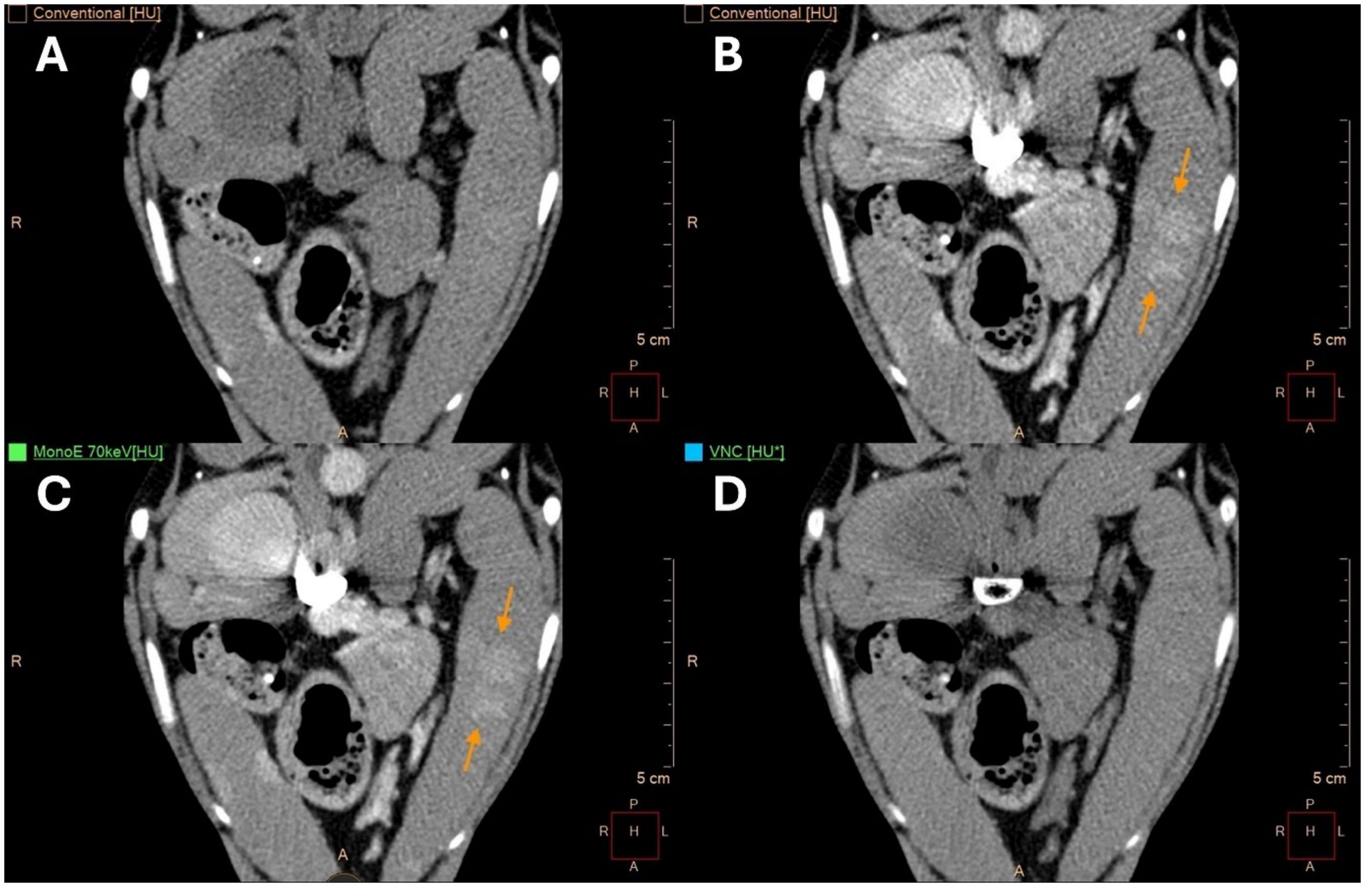
Transverse computed tomography (CT) images of the same patient shown in Figure 5. **(A)** Displays true unenhanced (TUE) images, **(B)** shows conventional images post-contrast, **(C)** features monoenergetic (MonoE) images at 70 keV post-contrast, and **(D)** includes virtual non-contrast (VNC) images. Orange arrows highlight one of the multifocal parenchymal lesions that became visible in the post-contrast images **(B,C)**. These lesions were accurately removed by the VNC algorithm (see **D**).

The second patient, who demonstrated larger discrepancies between VNC and TUE values, had several elongated mineralizations at the splenic hilus (Figure 5) and multiple central parenchymal lesions, both with and without mineralization. The hilar mineralizations were misinterpreted as areas of contrast enhancement and were incorrectly removed by the VNC algorithm (Figure 5D). The central parenchymal lesions became evident following contrast enhancement (e.g., orange arrows in Figures 6B,C). All ROIs that included mineralizations in this patient showed significant differences between VNC and TUE images. The discrepancies measured 53.2 HUs in the polygonal ROI of the largest mineralized parenchymal lesion, with 27 and 18.2 HUs in its periphery and 54.2 and 61.2 HUs in its center. The largest parenchymal lesion of this patient was not mineralized, and the polygonal ROI of this lesion exhibited a difference of less than 10 HUs. One of these non-mineralized parenchymal lesions is indicated by the orange arrows in Figure 6. The VNC algorithm accurately removed the contrast medium in these lesions (Figure 6D). This patient had extramedullary hematopoiesis and hemosiderosis on histopathology and was unique in having some lesions that displayed a moderate degree of mineralization.

The patients were categorized according to specific imaging characteristics of the splenic lesions to evaluate whether these influenced the performance of the VNC algorithm.

**Figure 7:**
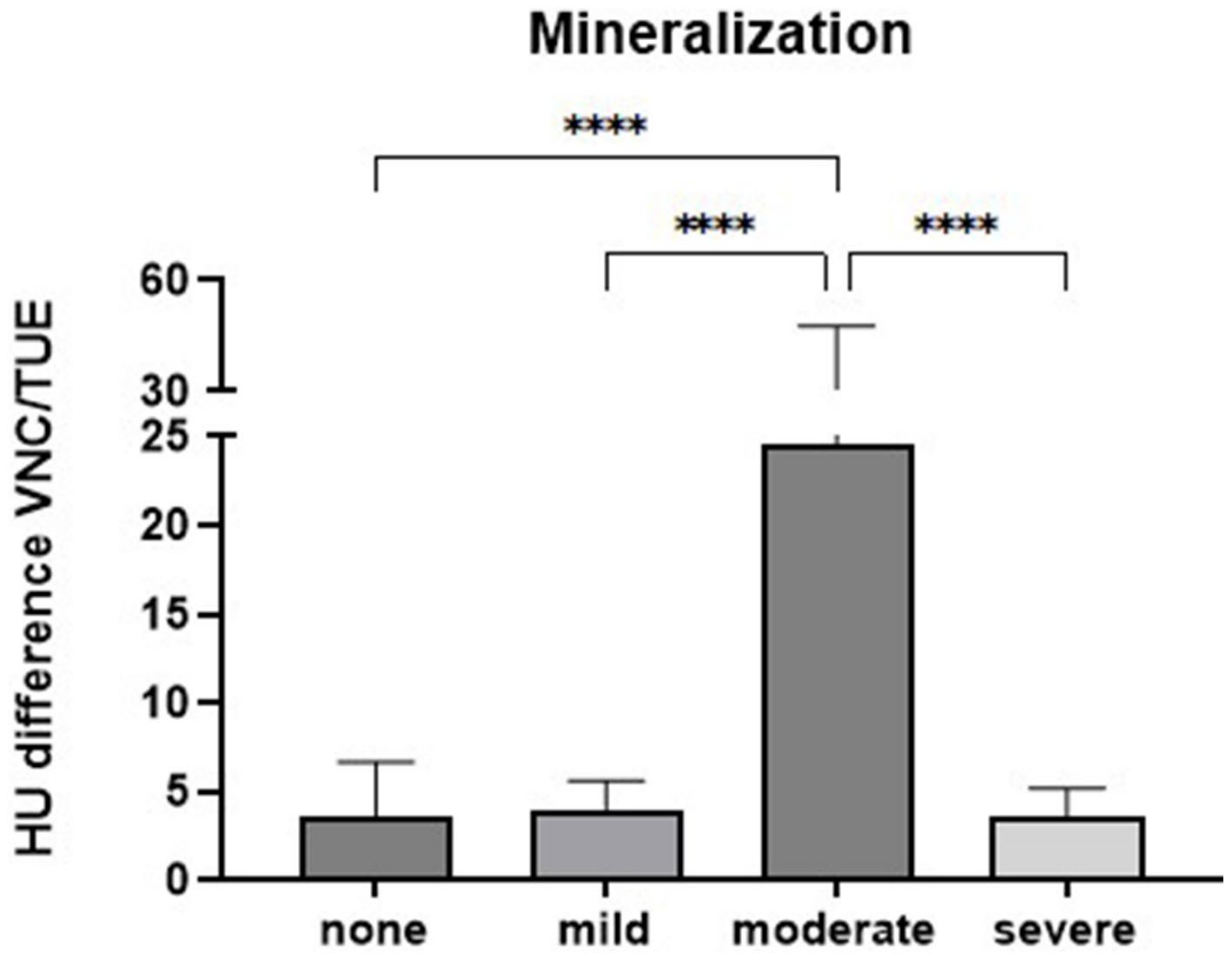
Comparison of the differences in Hounsfield units (HUs) between virtual non-contrast (VNC) and true unenhanced (TUE) images regarding the presence and degree of mineralization in the splenic lesions. The bars represent the mean values for each group with the standard deviation (SD) (****p < 0.0001).

The ordinary one-way ANOVA conducted to assess the influence of mineralization in lesions on the accuracy of the VNC technique produced a significant result, with *p* < 0.0001. The differences in HUs between VNC and TUE images for lesions with moderate mineralization (1) were considerably higher than those for lesions with no (26), mild (2), or severe (1) mineralization, with an adjusted p-value < 0.0001 in each comparison (Figure 7).

The comparison of differences in HU values between VNC and TUE images in patients with diffusely, focally, and multifocally altered spleens showed no significant difference, despite the patient with mineralized lesions being part of the multifocal group (p_ANOVA_ = 0.0768). The adjusted p-value for the comparison of diffusely altered to focally altered spleens was 0.9134; for diffusely altered to multifocally altered spleens, it was 0.6087; and for focally altered spleens compared to the multifocal altered group, it was 0.0750.

**Figure 8:**
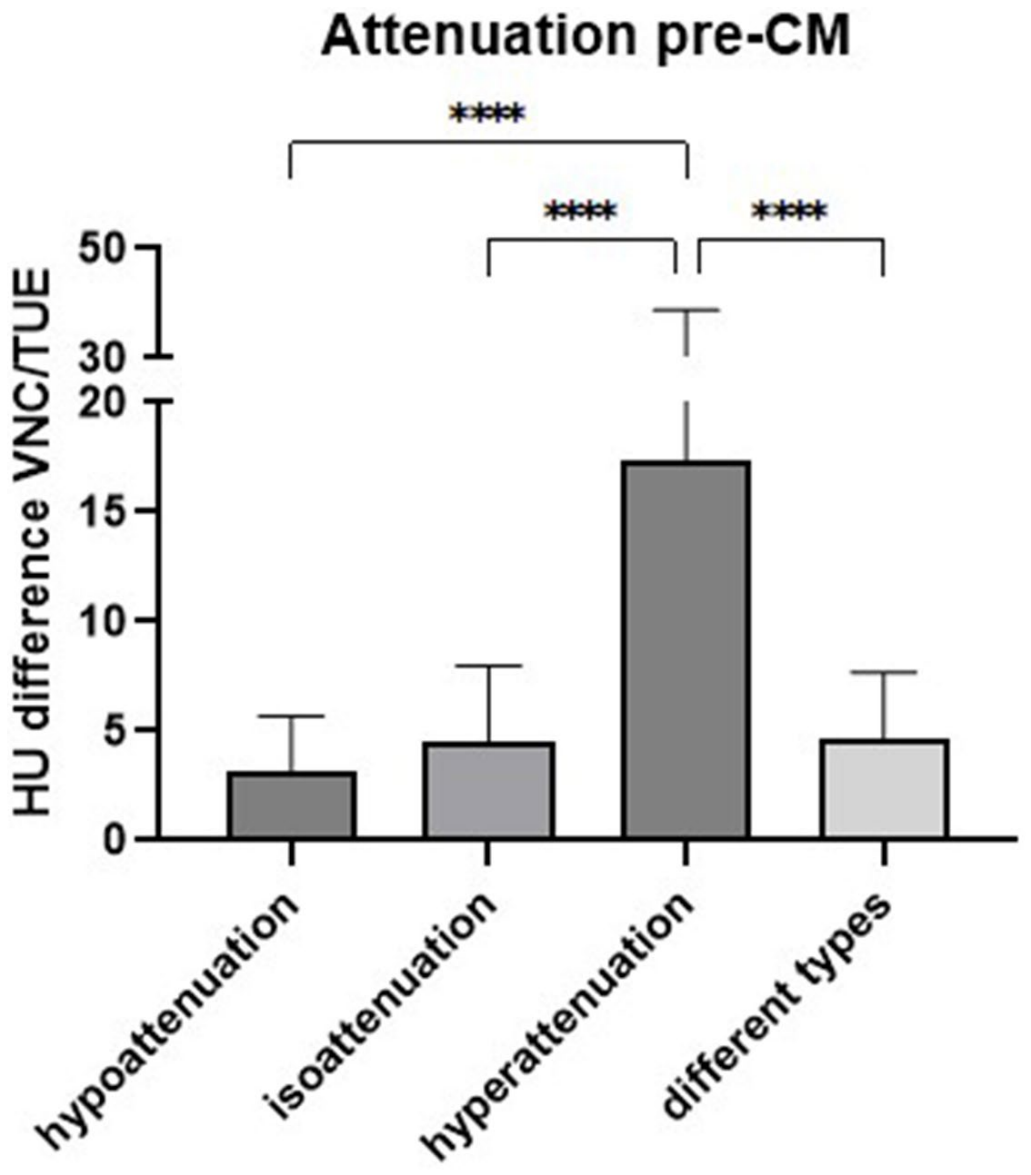
Comparison of the differences in Hounsfield units (HUs) values between virtual non-contrast (VNC) and true unenhanced (TUE) images of splenic lesions based on their attenuation relative to the surrounding tissue prior to the administration of contrast medium (CM). The bars represent the mean values for each group with the standard deviation (SD) (****p < 0.0001).

The comparison of patients regarding their type of attenuation before the administration of CM showed significant results (p_ANOVA_ < 0.0001). The largest difference in HUs between VNC and TUE images was noted in splenic lesions that were hyperattenuating compared to the surrounding tissue in TUE images, as observed in two patients. With an adjusted p-value < 0.0001 in each comparison, these results were significant when compared to lesions that were iso-attenuating (6), hypoattenuating (15), or lesions with various types of attenuation (5) in TUE images (Figure 8). Nevertheless, three of these four mean differences remained below the threshold of 10 HUs, which is considered negligible, while the mean difference for the group with hyperattenuating lesions was 17.37 HUs and included the patient with the highest differences due to incorrect measurement of its mineralized lesions. The two patients with diffusely altered spleens were excluded from this comparison due to the absence of normal-appearing parenchyma for comparison.

The comparison of various types of pathology showed a significantly higher mean difference in the HU values between TUE and VNC in patients with extramedullary hematopoiesis (4) compared to those with hemangiosarcoma (5), lymphoma (4), metastasis of anal-sac carcinoma (1), nodular hyperplasia (9), and hematoma (3) (p_ANOVA_ < 0.0001; adjusted *p*-values ranging from < 0.0001 to 0.0353). All other individual comparisons, including hemosiderosis (1), thrombus (1), and histopathologically unremarkable spleens (2), showed no significance. A comparison of malignant and benign lesions revealed no statistical significance.

All results of the multiple comparisons, including the presence of cavitation, the type of attenuation of the splenic lesions post-CM compared to the surrounding tissue, the type of enhancement pattern, and the degree of enhancement, are presented in Supplementary Table S1.

An unpaired t-test showed no significant differences in HU values between TUE and VNC images within regions of interest located at the center compared to the periphery of the focal lesions (*p* = 0.1810), irrespective of the presence of mineralization (*p* = 0.2215).

### 3.5 Subjective assessment

The quality of the VNC images was slightly superior to that of the TUE images, receiving an average score of 4.07 (SD ± 0.25) on a five-point Likert scale. In two patients the image quality of VNC images was assessed as much better (score 5) than the image quality of TUE images. All other patients (28/30) received a score of 4.

The iodine subtraction in the pancreas, gallbladder, spinal musculature, liver, spleen, and main lesion was nearly complete, with an average score of 4.37 points (SD ± 0.30). The spinal musculature achieved the highest score, at 4.97 points (SD ± 0.18). Overall, the splenic tissue scored 4.13 points (SD ± 0.72), while the main lesions of the spleen (excluding the two diffuse pathologies) received a minimum score of 3.4 points (SD ± 1.2).

## 4 Discussion

The findings of this study support our hypothesis that HUs in VNC images demonstrate high reliability compared to those from TUE images in canine splenic pathologies. No significant difference was observed for a cut-off value of < 15 HUs in terms of HU values of VNC compared to TUE images in reference organs and affected spleens. An acceptable cut-off value of ≤ 15 HUs was achieved in 98.0% of 305 ROIs, with 93.1% showing a difference of less than 10 HUs, and 66.2% remaining below the limit of a 5 HUs difference. These results are slightly better than those reported for healthy dogs using the same settings (19), where the differences were ≤ 15 HUs in 91.61%, ≤ 10 HUs in 78.67%, and ≤ 5 HUs in 50.87% of all compared ROIs, confirming the assumption that VNC performs accurately in structurally altered splenic tissue.

The VNC technique aims to be equivalent to TUE images. It does not yet improve diagnostic capability or accuracy in characterizing splenic pathologies via CT, since conventional CT already lacks the ability to distinguish benign from malignant conditions of the spleen (2–5, 7). Although spectral CT has the potential to enhance diagnostic abilities through additional algorithms, such as quantification of perfusion with perfusion mapping, this remains the aim of future studies.

The equivalence of VNC to TUE images offers potential to omit the native scan in the future. This approach has already been implemented for various pathologies in human medicine (10, 12, 17, 22–28). Further research is required to gather more information on the use of VNC regarding different organ pathologies. In human medicine, the VNC technique has proven useful in dose calculation for radiotherapy planning. Contrast medium is used in radiation therapy planning to improve visualisation of relevant structures, but it may cause dose calculation errors by inaccurately converting Hounsfield units to electron density. The VNC scan is utilized in these patients to determine tissue electron density, thereby improving the accuracy of radiation dose calculations (31). This approach has the same impact on our veterinary patients receiving radiation therapy.

The cut-off values for the differences in HUs between the two series came from human medicine studies (13–15, 17, 32). Ananthakrishnan et al. (14) and Sauter et al. (13), for instance, defined a difference in HU values between TUE images and VNC images of 10 to 15 HUs as acceptable, while differences below 10 HUs were considered negligible. This definition is based on the human eye’s ability to perceive only a change of 6% in grayscale (32). Thus, in a standard abdominal CT window with a width of 400 HUs, spanning from black (−150 HUs) to white (+250 HUs), there are 398 different shades of gray present; however, a change of only 24 shades is discernible to a human observer. The cut-off value of ≤ 5 HUs was also included to assess and illustrate the potential and accuracy of VNC images.

No significant differences were found in the comparison of HU-values between VNC and TUE images for different types of lesions (diffuse, focal, and multifocal) based on their imaging characteristics. However, mineralized lesions seemed to influence the calculation of VNC images. In one patient with multiple splenic lesions that exhibited moderate mineralization, the statistical analysis indicated significantly greater differences in HU-values compared to patients without (26) or with mild (2) or severe (1) mineralization. The HU-values of these lesions ranged from 88.7 to 137.5 in TUE and from 67.9 to 76.3 in VNC, suggesting that mineralized lesions could potentially be misinterpreted, for example, as hemorrhage. This may present a significant drawback in the interpretation of malignant neoplasia. The small number of mineralized lesions in our study limits the validity of statistical interpretation and should be regarded as a tendency needing further evaluation in future studies, particularly in cases where the detection of mineralization is crucial for decision-making (e.g., urolithiasis). The decreased performance of the VNC algorithm in mineralized lesions might be improved by the supplementary use of atomic number mapping, another spectral CT technique, and should be investigated in future studies (24).

Pre-contrast hyperattenuating lesions were rare, occurring in only two cases. However, these lesions showed the greatest difference in HUs between TUE and VNC when compared to pre-contrast iso- or hypoattenuating lesions. A hyperattenuating splenic lesion in TUE images may indicate acute hemorrhage (with normal splenic tissue ranging from 40 to 60 HUs and coagulated blood between 60 and 80 HUs) or early mineralization (> 100 HUs). Further studies are needed to determine whether pre-contrast hyperattenuating splenic lesions pose a challenge for the VNC algorithm.

As the VNC algorithm has known issues in subtracting contrast medium from smaller hepatic vessels (17), we were interested in evaluating the performance of the VNC images at the periphery of malignant lesions, where neovascularization is suspected. No statistically significant differences were observed in the values related to the periphery of neoplastic and non-neoplastic lesions, or between the periphery and center of these lesions.

Although there were some statistically significant differences in the various imaging characteristics of splenic lesions, the differences between the HUs in VNC and TUE images all fell below the 15 HUs threshold, except for the previously mentioned mineralised lesion. This suggests that these changes are not discernible to the human eye in a standard soft tissue window and are, therefore, likely not clinically relevant.

The comparison of the various types of pathology revealed a significantly higher mean difference in HU values between VNC and TUE images in patients with extramedullary hematopoiesis than in those with hemangiosarcoma, lymphoma, carcinoma metastasis, nodular hypoplasia, and hematoma. However, this group was also the largest, including the patient with significant differences in mineralized lesions, as mentioned previously.

The specific cause of the slightly decreased performance of VNC in extramedullary hematopoiesis remains unknown. Possible explanations might relate to the pathological changes associated with this condition, including the reactivation of pluripotent stem cells, hyperplasia of hematopoietic cells, and increased blood flow, which could alter the two-material water-iodine model. Iodine mapping, another spectral CT technique, may serve as a valuable supplement in these cases to better quantify tissue perfusion (12, 24). Future research could explore different spectral maps to determine if the limitations of the VNC method can be addressed. Nonetheless, 81.5% of ROIs in cases of extramedullary hematopoiesis had differences below the threshold of < 15 HUs.

Unfortunately, the various malignant pathologies, which comprise a heterogeneous group, could not be further statistically analysed due to the limited number of cases.

The subjective image quality of spectral CT images was superior to that of conventional CT images, proving our second hypothesis. Although the average score for the splenic main lesions in patients with focal and multifocal lesions was 3.4 points on a 5-point Likert scale, the subjective analysis indicated that the overall image quality of VNC is slightly better than that of TUE, with an average score of 4.07 points. Typically, the image quality of spectral detector CT images surpasses that of conventional images; however, we observed that some streak artefacts, caused by the incomplete mixing of the contrast medium with blood in larger vessels, diminish image quality in post-contrast images. These artefacts persist in VNC images, even though the contrast medium has been effectively removed from the images (see Figure 6D).

Additionally, the iodine subtraction observed in the pancreas, gallbladder, spinal muscle, liver, spleen, and the main lesion was nearly complete, with an average score of 4.37 points. Compared to other studies in human and veterinary medicine, this result is consistent with the VNC images (13, 15, 19).

One main limitation of this study is the small number of patients and the heterogeneous groups of pathologies. VNC performed well across all types of lesions; however, the distribution of these groups was inhomogeneous, with only two diffusely altered spleens. Diffuse splenic pathologies without splenic enlargement may have been overlooked initially when selecting cases. Further studies with a larger cohort of patients are needed to validate our findings.

Only the main findings of the histopathological workup were considered when grouping the patients to evaluate their influence on the accuracy of VNC values. As noted in the results, hemosiderosis and extramedullary hematopoiesis were common concomitant conditions. This might explain why we frequently observed different imaging characteristics for various lesions in multifocal pathologies. We had two patients in whom the splenic pathology was diagnosed by Tru-Cut biopsies. These biopsies were taken under ultrasound guidance. Since there are known differences between ultrasound and CT examinations regarding splenic pathologies, we could not guarantee that the lesions examined by histopathology corresponded to the lesions visualized on CT images. Additionally, in all other cases where the entire spleen was evaluated in pathology, we cannot be certain that the examiner assessed the CT-detected lesions.

The characterization of splenic lesions was conducted solely during the venous phase, and the additional information from the arterial phase may have been overlooked. The efficacy of the VNC technique in both the arterial and portal venous phases has been assessed in human medicine through various studies (13, 17, 24). A study from 2018 by Sauter et al. (13) demonstrated promising results for VNC images reconstructed from both the arterial and portal venous phases. Another study from 2020 in human medicine (17) revealed no significant differences when comparing true unenhanced images with VNC images from the arterial phase and VNC images from the venous phase. Although we did not include the arterial phase in our study, further investigations focusing on specific pathologies may need to incorporate these series to ascertain the impact of the different contrast phases.

## 5 Conclusion

Comparing VNC images with TUE images showed excellent agreement in splenic pathologies. The quality of spectral-based image reconstructions exceeded that of conventional native scans.

Omitting the native scan in a clinical setting remains impossible, as the VNC technique must validate its performance for pathologies of various abdominal and thoracic organs. Mineralized lesions, in particular, may pose a challenge for the algorithm, necessitating further research to determine whether this potential issue affects decision-making.

## Data Availability

The raw data supporting the conclusions of this article will be made available by the authors, without undue reservation.

## Glossary

ANOVA: Analysis of variance
AVG: Alkje van Gemmeren
CT: Computed tomography
CE: contrast-enhanced
CM: contrast medium
DECT: Dual-Energy-CT
DPCT: Dual-Phase-CT
ECVDI: European College of Veterinary Diagnostic Imaging
ECVP: European College of Veterinary Pathologists
eV: Electronvolt
e.g: exempli gratia / for example
HU: Hounsfield Unit
ICM: iodine-based contrast medium
keV: kilo-electronvolt
KM: Kristina Merhof
LC: Lydia Claußen
mAs: Milliampere-seconds
MonoE: monoenergetic
PL: Philipp Lietz
ROI: Region of Interest
SBI: spectral based images
SD: standard deviation
SDCT: Spectral-detector CT
TUE: true unenhanced → conventional non-contrast images
VNC: Virtual non-contrast

## References

[1] Burti S Zotti A Bonsembiante F Contiero B Banzato T. A machine learning-based approach for classification of focal splenic lesions based on their CT features. Front Vet Sci. (2022) 9:872618. doi: 10.3389/fvets.2022.872618 35585859 PMC9108536

[2] Burti S Zotti A Contiero B Banzato T. Computed tomography features for differentiating malignant and benign focal liver lesions in dogs: a meta-analysis. Vet J. (2021) 278:105773. Available from:. doi: 10.1016/j.tvjl.2021.105773, PMID: 34742915

[3] Jones ID Lamb CR Drees R Priestnall SL Mantis P. Associations between dual-phase computed tomography features and histopathologic diagnoses in 52 dogs with hepatic or splenic masses. Vet Radiol Ultrasound. (2016) 57:144–53. doi: 10.1111/vru.12336 26763951

[4] Jones ID Daniels AD Lara-Garcia A Peters LM Mantis P. Computed tomographic findings in 12 cases of canine multi-centric lymphoma with splenic and hepatic involvement. J Small Anim Pract. (2017) 58:622–8. doi: 10.1111/jsap.12714, PMID: 28762504

[5] Hughes JR Szladovits B Drees R. Abdominal CT evaluation of the liver and spleen for staging mast cell tumors in dogs yields nonspecific results. Vet Radiol Ultrasound. (2019) 60:306–15. doi: 10.1111/vru.12717, PMID: 30786323

[6] Mesquita L Finotello R Ferreira A Maddox T. Comparison between computed tomographic and ultrasonographic findings of the liver and spleen in dogs with confirmed hepatic or splenic lymphoma involvement. Vet Rec. (2022) 190:e780. doi: 10.1002/vetr.780, PMID: 34352128

[7] Cordella A Caldin M Bertolini G. Splenic extramedullary hematopoiesis in dogs is frequently detected on multiphase multidetector-row CT as hypervascular nodules. Vet Radiol Ultrasound. (2020) 61:512–8. doi: 10.1111/vru.12872, PMID: 32579754

[8] Fife WD Samii VF Drost T Mattoon JS Hoshaw-Woodard S. Comparison between malignant and nonmalignant splenic masses in dogs using contrast-enhanced computed tomography. Vet Radiol Ultrasound. (2004) 45:289–97. doi: 10.1111/j.1740-8261.2004.04054.x, PMID: 15373250

[9] Kutara K Seki M Ishigaki K Teshima K Ishikawa C Kagawa Y. Triple-phase helical computed tomography in dogs with solid splenic masses. J Vet Med Sci. (2017) 79:1870–7. doi: 10.1292/jvms.17-0253 28993600 PMC5709567

[10] Agrawal MD Pinho DF Kulkarni NM Hahn PF Guimaraes AR Sahani DV. Oncologic applications of dual- energy CT in the abdomen. Radiographics. (2014) 34:589–612. doi: 10.1148/rg.343135041, PMID: 24819783

[11] Connolly MJ McInnes MDF El-Khodary M McGrath TA Schieda N. Diagnostic accuracy of virtual non-contrast enhanced dual-energy CT for diagnosis of adrenal adenoma: a systematic review and meta-analysis. Eur Radiol. (2017) 27:4324–35. doi: 10.1007/s00330-017-4785-0 28289937

[12] Sauerbeck J Adam G Meyer M. Spectral CT in oncology. RöFo. (2023) 195:21–9. doi: 10.1055/a-1902-9949 36167316

[13] Sauter AP Muenzel D Dangelmaier J Braren R Pfeiffer F Rummeny EJ. Dual-layer spectral computed tomography: virtual non-contrast in comparison to true non-contrast images. Eur J Radiol. (2018) 104:108–14. doi: 10.1016/j.ejrad.2018.05.007, PMID: 29857855

[14] Ananthakrishnan L Rajiah P Ahn R Rassouli N Xi Y Soesbe TC. Spectral detector CT-derived virtual non-contrast images: comparison of attenuation values with unenhanced CT. Abdom Radiol. (2017) 42:702–9. doi: 10.1007/s00261-016-1036-9, PMID: 28084546

[15] Jamali S Michoux N Coche E Dragean CA. Virtual unenhanced phase with spectral dual-energy CT: is it an alternative to conventional true unenhanced phase for abdominal tissues? Diagn Interv Imaging. (2019) 100:503–11. doi: 10.1016/j.diii.2019.04.007, PMID: 31155514

[16] Kaufmann S Sauter A Spira D Gatidis S Ketelsen D Heuschmid M. Tin-filter enhanced dual-energy-CT. Acad Radiol. (2013) 20:596–603. doi: 10.1016/j.acra.2013.01.010, PMID: 23490736

[17] Laukamp KR Ho V Obmann VC Herrmann K Gupta A Borggrefe J. Virtual non-contrast for evaluation of liver parenchyma and vessels: results from 25 patients using multi-phase spectral-detector CT. Acta Radiol. (2020) 61:1143–52. doi: 10.1177/0284185119893094, PMID: 31856581

[18] Goo HW Goo JM. Dual-energy CT: new horizon in medical imaging. Korean J Radiol. (2017) 18:555–69. doi: 10.3348/kjr.2017.18.4.555 28670151 PMC5447632

[19] Lietz P Brüntgens M Wang-Leandro A Volk HA Meller S Merhof K. Virtual non-contrast images of detector-based spectral computed tomography in dogs: a promising alternative to true non-contrast images in veterinary medicine. Front Vet Sci. (2023) 10:1251535. doi: 10.3389/fvets.2023.1251535 38105773 PMC10722308

[20] Germonpré J Vandekerckhove LMJ Raes E Chiers K Jans L Vanderperren K. Post-mortem feasibility of dual-energy computed tomography in the detection of bone edema-like lesions in the equine foot: a proof of concept. Front Vet Sci. (2024) 10:1201017. doi: 10.3389/fvets.2023.1201017 38249561 PMC10797750

[21] Mikić M Lietz P Meller S Pees M Merhof K. Evaluation of virtual non-contrast detector-based spectral CT images in comparison to true unenhanced images in 20 rabbits. Front. Vet. Sci. (2025) 12:1521986. doi: 10.3389/fvets.2025.1521986 40151564 PMC11947665

[22] Laroia ST Bhadoria AS Venigalla Y Chibber GK Bihari C Rastogi A. Role of dual energy spectral computed tomography in characterization of hepatocellular carcinoma: initial experience from a tertiary liver care institute. Eur J Radiol Open. (2016) 3:162–71. doi: 10.1016/j.ejro.2016.05.007, PMID: 27504474 PMC4968142

[23] Yu Y Guo L Hu C Chen K. Spectral CT imaging in the differential diagnosis of necrotic hepatocellular carcinoma and hepatic abscess. Clin Radiol. (2014) 69:e517–24. doi: 10.1016/j.crad.2014.08.018, PMID: 25248290

[24] Adam SZ Rabinowich A Kessner R Blachar A. Spectral CT of the abdomen: where are we now? Insights Imaging. (2021) 12:138. doi: 10.1186/s13244-021-01082-7, PMID: 34580788 PMC8476679

[25] Anzidei M Di Martino M Sacconi B Saba L Boni F Zaccagna F. Evaluation of image quality, radiation dose and diagnostic performance of dual-energy CT datasets in patients with hepatocellular carcinoma. Clin Radiol. (2015) 70:966–73. doi: 10.1016/j.crad.2015.05.003, PMID: 26095726

[26] Graser A Johnson TRC Hecht EM Becker CR Leidecker C Staehler M. Dual-energy CT in patients suspected of having renal masses: can virtual nonenhanced images replace true nonenhanced images? Radiology. (2009) 252:433–40. doi: 10.1148/radiol.2522080557 19487466

[27] Jing M Sun J Xi H Liu Z Zhang S Deng L. Abdominal virtual non-contrast images derived from energy spectrum CT to evaluate chemotherapy-related fatty liver disease. Quant Imaging Med Surg. (2023) 13:669–81. doi: 10.21037/qims-22-742, PMID: 36819287 PMC9929393

[28] Szablics FÉ Bérczi Á Csőre J Borzsák S Szentiványi A Kiss M. Csobay-Novák C Virtual non-contrast reconstructions derived from dual-energy CTA scans in peripheral arterial disease: comparison with true non-contrast images and impact on radiation dose. J Clin Med. (2025) 14:5571. doi: 10.3390/jcm14155571 40807191 PMC12346895

[29] Ma G Han D Dang S Yu N Yang Q Yang C. Replacing true unenhanced imaging in renal carcinoma with virtual unenhanced images in dual-energy spectral CT: a feasibility study. Clin Radiol. (2021) 76:81.e21–7. doi: 10.1016/j.crad.2020.08.026, PMID: 32993881

[30] Bucolo GM Ascenti V Barbera S Fontana F Aricò FM Piacentino F. Virtual non-contrast spectral CT in renal masses: is it time to discard conventional unenhanced phase? J Clin Med. (2023) 12:718. doi: 10.3390/jcm12144718, PMID: 37510833 PMC10380803

[31] Saito M Saito T. A simple algorithm to derive virtual non-contrast electron density from dual-energy computed tomography data for radiotherapy treatment planning. Med Phys. (2025) 52:3107–16. doi: 10.1002/mp.17648, PMID: 39865311 PMC12082761

[32] Malik P Vidyarthi A. Stacked deep model-based classification of the multiclass brain hemorrhages in CT scans. Int J Imaging Syst Technol. (2024) 34:1–16. doi: 10.1002/ima.22955

