# Supplementary material for: Spectral detector computed tomography imaging of histologically confirmed splenic pathologies in 30 canine patients: a comparison of virtual non-contrast images and true unenhanced images
Source: Front Vet Sci. 2025 Sep 25;12:1645439. doi: 10.3389/fvets.2025.1645439 (PMC12511805; doi:10.3389/fvets.2025.1645439)
Supplement: Supplementary file 1 [file Table_1.DOCX]

**Supplementary Table 1:** Results of the multiple comparisons of the differences in Hounsfield Units (HUs) between virtual non-contrast (VNC) and true unenhanced (TUE) images categorized according to specific imaging characteristics of the splenic lesions. A one-way analysis of variance (ANOVA) test was performed, including Tukey`s multiple comparisons test for p-value correction. A p-value of less than 0.05 has been deemed significant.
CI: Confidence interval; CM: contrast medium; EMH: Extramedullary hematopoiesis; WF: Without findings; vs: versus; ns = not significant; *: significant; **: highly significant; ***: very highly significant; ****: extremely significant

| **Imaging characteristics of splenic lesions** | **Comparison** | **Mean difference in HU-values between VNC and TUE** | **CI (95%) of difference** | **Adjusted p-value** | **Level of significance** |
| --- | --- | --- | --- | --- | --- |
| Type of lesion  (p_ANOVA_ = 0.0768) | Diffusely altered spleen vs. focal splenic lesion | 1.075 | -5.197 to 7.346 | 0.9134 | ns |
|  | Diffusely altered spleen vs. multifocal splenic lesion | -2.288 | -7.978 to 3.402 | 0.6087 | ns |
|  | Focal splenic lesion vs. multifocal splenic lesion | -3.362 | -6.985 to 0.2605 | 0.0750 | ns |
| Mineralization of lesions (p_ANOVA_ < 0.0001) | None vs. mild mineralization | -0.3903 | -5,722 to 4,941 | 0.9976 | ns |
|  | None vs. moderate mineralization | -20.84 | -26,17 to -15,51 | <0.0001 | **** |
|  | None vs. severe mineralization | 0.0297 | -5,302 to 5,361 | >0.999 | ns |
|  | Mild vs. moderate mineralization | -20.45 | -27,71 to -13,19 | <0.0001 | **** |
|  | Mild vs. severe mineralization | 0.42 | -6,844 to 7,684 | 0.9988 | ns |
|  | Moderate vs. severe mineralization | 20.87 | 13,61 to 28,13 | <0.0001 | **** |
| Cavitation of lesions (p_ANOVA_ = 0.0179) | None vs. cavitation in one lesion | 3.895 | 0,4147 to 7,376 | 0.0241 | * |
|  | None vs. cavitation in several lesions | 3.218 | -1,337 to 7,772 | 0.2193 | ns |
|  | Cavitation in one lesion vs. cavitation in several lesions | -0.6775 | -5,765 to 4,410 | 0.9468 | ns |
| Attenuation of lesions pre-CM compared to the surrounding tissue  (p_ANOVA_ < 0.0001) | Hypoattenuation vs. isoattenuation | -1.336 | -5,526 to 2,854 | 0.8407 | ns |
|  | Hypoattenuation vs. hyperattenuation | -14.2 | -19,49 to -8,912 | < 0.0001 | **** |
|  | Hypoattenuation vs. different types of attenuation | -1.447 | -5,637 to 2,743 | 0.8060 | ns |
|  | Isoattenuation vs. hyperattenuation | -12.87 | -18,91 to -6,824 | < 0.0001 | **** |
|  | Isoattenuation vs. different types of attenuation | -0.1111 | -5,218 to 4,996 | > 0.9999 | ns |
|  | Hyperattenuation vs. different types of attenuation | 12.76 | 6,712 to 18,80 | < 0.0001 | **** |
| Attenuation of lesions post-CM compared to the surrounding tissue (p_ANOVA_ = 00.15) | Hypoattenuation vs. hyperattenuation | -5.578 | -9,393 to -1,764 | 0.0020 | ** |
|  | Hypoattenuation vs. different types of attenuation | -1.414 | -5,399 to 2,571 | 0.6786 | ns |
|  | Hyperattenuation vs. different types of attenuation | 4.164 | 0,4438 to 7,885 | 0.0241 | * |
| Enhancement pattern of splenic lesions (p_ANOVA_ < 0.0001) | Homogeneous vs. heterogeneous enhancement pattern | 6.532 | 3,032 to 10,03 | < 0.0001 | **** |
|  | Homogeneous vs. different types of enhancement pattern | 4.802 | 0,8133 to 8,790 | 0.0137 | * |
|  | Heterogeneous vs. different types of enhancement pattern | -1.730 | -5,310 to 1,851 | 0.4890 | ns |
| Degree of enhancement of the lesions  (p_ANOVA_ = 0.0030) | Mild vs. moderate degree of enhancement | -5.003 | -9,323 to -0,6831 | 0.0161 | * |
|  | Mild vs. severe degree of enhancement | -0.9071 | -6,319 to 4,505 | 0.9723 | ns |
|  | Mild vs. different degrees of enhancement in different lesions | 0.1588 | -4,765 to 5,082 | 0.9998 | ns |
|  | Moderate vs. Severe degree of enhancement | 4.096 | -0,7736 to 8,965 | 0.1320 | ns |
|  | Moderate vs. different degrees of enhancement in different lesions | 5.162 | 0,8419 to 9,482 | 0.0121 | * |
|  | Severe vs. different degrees of enhancement in different lesions | 1.066 | -4,346 to 6,478 | 0.9562 | ns |
| Histopathological diagnosis (p_ANOVA_ < 0.0001) | Hemangiosarcoma vs. Lymphoma | -0,4563 | -6,994 to 6,082 | >0,9999 | ns |
|  | Hemangiosarcoma vs. Metastatic carcinoma | 1,712 | -9,496 to 12,92 | >0,9999 | ns |
|  | Hemangiosarcoma vs. Nodular hyperplasia | -0,7240 | -6,328 to 4,880 | >0,9999 | ns |
|  | Hemangiosarcoma vs. Hematoma | -1,556 | -9,379 to 6,267 | 0,9994 | ns |
|  | Hemangiosarcoma vs. EMH | -10,28 | -16,63 to -3,931 | <0,0001 | **** |
|  | Hemangiosarcoma vs. Thrombus | -0,5730 | -12,89 to 11,75 | >0,9999 | ns |
|  | Hemangiosarcoma vs. Hemosiderosis | -4,048 | -16,37 to 8,272 | 0,9819 | ns |
|  | Hemangiosarcoma vs. WF | -0,2051 | -9,988 to 9,578 | >0,9999 | ns |
|  | Lymphoma vs. Metastatic carcinoma | 2,168 | -9,079 to 13,42 | 0,9996 | ns |
|  | Lymphoma vs. Nodular hyperplasia | -0,2677 | -5,949 to 5,414 | >0,9999 | ns |
|  | Lymphoma vs. Hematoma | -1,099 | -8,978 to 6,779 | >0,9999 | ns |
|  | Lymphoma vs. EMH | -9,825 | -16,24 to -3,407 | 0,0001 | *** |
|  | Lymphoma vs. Thrombus | -0,1167 | -12,47 to 12,24 | >0,9999 | ns |
|  | Lymphoma vs. Hemosiderosis | -3,592 | -15,95 to 8,764 | 0,9918 | ns |
|  | Lymphoma vs. WF | 0,2512 | -9,577 to 10,08 | >0,9999 | ns |
|  | Metastatic carcinoma vs. Nodular hyperplasia | -2,436 | -13,17 to 8,295 | 0,9985 | ns |
|  | Metastatic carcinoma vs. Hematoma | -3,268 | -15,31 to 8,772 | 0,9949 | ns |
|  | Metastatic carcinoma vs. EMH | -11,99 | -23,13 to -0,8547 | 0,0245 | * |
|  | Metastatic carcinoma vs. Thrombus | -2,285 | -17,63 to 13,06 | >0,9999 | ns |
|  | Metastatic carcinoma vs. Hemosiderosis | -5,760 | -21,11 to 9,587 | 0,9593 | ns |
|  | Metastatic carcinoma vs. WF | -1,917 | -15,31 to 11,48 | >0,9999 | ns |
|  | Nodular hyperplasia vs. Hematoma | -0,8317 | -7,954 to 6,291 | >0,9999 | ns |
|  | Nodular hyperplasia vs. EMH | -9,557 | -15,02 to -4,093 | <0,0001 | **** |
|  | Nodular hyperplasia vs. Thrombus | 0,1510 | -11,74 to 12,04 | >0,9999 | ns |
|  | Nodular hyperplasia vs. Hemosiderosis | -3,324 | -15,21 to 8,564 | 0,9937 | ns |
|  | Nodular hyperplasia vs. WF | 0,5189 | -8,714 to 9,752 | >0,9999 | ns |
|  | Hematoma vs. EMH | -8,726 | -16,45 to -1,002 | 0,0144 | * |
|  | Hematoma vs. Thrombus | 0,9827 | -12,10 to 14,06 | >0,9999 | ns |
|  | Hematoma vs. Hemosiderosis | -2,492 | -15,57 to 10,59 | 0,9996 | ns |
|  | Hematoma vs. WF | 1,351 | -9,375 to 12,08 | >0,9999 | ns |
|  | EMH vs. Thrombus | 9,708 | -2,549 to 21,97 | 0,2438 | ns |
|  | EMH vs. Hemosiderosis | 6,233 | -6,024 to 18,49 | 0,8031 | ns |
|  | EMH vs. WF | 10,08 | 0,3726 to 19,78 | 0,0353 | * |
|  | Thrombus vs. Hemosiderosis | -3,475 | -19,65 to 12,70 | 0,9990 | ns |
|  | Thrombus vs. WF | 0,3679 | -13,97 to 14,71 | >0,9999 | ns |
|  | Hemosiderosis vs. WF | 3,843 | -10,50 to 18,18 | 0,9953 | ns |
